# The Association of the Prolonged Use of Personal Protective Equipment and Face Mask During COVID-19 Pandemic With Various Dermatologic Disease Manifestations: A Systematic Review

**DOI:** 10.7759/cureus.16544

**Published:** 2021-07-21

**Authors:** Ghassan M Barnawi, Azhar M Barnawi, Sahal Samarkandy

**Affiliations:** 1 Dermatology, College of Medicine at King Saud bin Abdulaziz University for Health Sciences, King Abdulaziz Medical City, Jeddah, SAU; 2 Family Medicine, Armed Forces Hospital, Prince Mansour Military Hospital for Community Medicine, Taif, SAU

**Keywords:** dermal manifestations, covid-19, ppe, long duration, impact

## Abstract

COVID-19 is a novel coronavirus that represents a great global health concern. It is transmitted between individuals through respiratory particles, and as there is no established effective treatment currently for the virus, it is necessary to use protective strategies such as face masks. Healthcare providers and individuals serving outdoors are required to work for long durations wearing personal protective equipment (PPE). Wearing such protective equipment may have short- or long-term effects on the skin health of these individuals. We aim to assess the prevalence and types of dermatological manifestations associated with wearing PPE for a long time during the COVID-19 pandemic period by reviewing the previous studies conducted on this subject.

The medical literature, including the databases of PubMed and Google Scholar, from 2019 to 2021 was explored. The search terms included a combination of "Impact of PPE and dermatological outcome," "Wearing PPE for a long time and dermatological manifestations," "Face mask-wearing and dermatological complications," and "Wearing PPE and dermatological manifestations." The inclusion criteria are original full-text articles that reported the association of wearing PPE and dermatological manifestations and outcomes.

A total of 70 articles were obtained, among which only 10 articles were eligible for the inclusion criteria. These 10 studies included a total number of 7,643 participants and covered different countries of the world. The extracted data were summarized in a table to facilitate the collection of the important findings.

Dermatological complications are frequently reported in people wearing PPE and face masks, especially the ones caused due to PPE among healthcare providers as they tend to use these protective measures for longer durations.

## Introduction and background

The novel strain of coronavirus was first reported in Wuhan, China, in late November 2019 [1]. Now known as COVID-19, it has progressed to become a global public health emergency [2]. Symptoms typically include fever, a sore throat, shortness of breath, headaches, and coughing; however, asymptomatic patients are also known to exist [2]. Until May 2020, there was no approved vaccine for COVID-19 [3], and although they do now exist, active treatment is still evolving, revolving mostly around supportive care such as supplementation of oxygen and maintaining continuous positive airway pressure [4]. Transmission between humans occurs at high rates, spreading via droplet particulates or physical contact with a contaminated surface, causing all mundane social interactions from breathing to conversing a pathway for infection. [3]. For this reason, it was deemed necessary to enforce preventive strategies at the respiratory level, the most widespread being the use of face masks as a form of personal protective equipment (PPE) when within 2 m of another person or in enclosed spaces [5]. In high contagion areas, the forms of PPE required are more extreme, with some offering protection to the nose, mouth, ears, eyes, hands, and any other exposed areas such as the head [6]. In the majority of cases, PPE is restricted to respiratory protective equipment, most commonly a surgical or cotton mask, although some visors are available [6]. These have been shown to offer the highest preventive capabilities by serving as an immediate barrier between the respiratory tract and the surrounding environment [7]. The efficacy of face masks is highly reliant on how well it fits the contours of each individual face, the material, and the health of the individual in question [7]. This has led to increased popularity in respirator masks, which are being designed to create an adaptable facial seal, while also being recyclable - a definite improvement in traditional surgical masks [7].

During the COVID-19 pandemic, both healthcare workers (HCWs) and the general population have had a legal obligation to wear PPE in public at all times, be it at work or in transit. This has led to some unforeseen consequences such as an apparent increased susceptibility to adverse skin reactions [8]. This is so far attributed to factors related to moisture, excessive sweating, and friction caused by near-constant exposure to the fabrics, synthetic or otherwise, of any PPE. In addition, evidence suggests that the higher performance filtration PPEs are associated with increased dermatological conditions when compared to the traditional medical mask [9].

The epidemiology of skin diseases related to masks is yet to be widely studied, with the majority of such work being consigned to simple case reports or small local studies [10]. However, as the medical profession demands a near-constant use of respiratory PPE at the very least and for considerable periods of time, it has become necessary to understand and assess the various dermatological manifestations and adverse skin reactions that are being increasingly reported with the prolonged use of PPE. With this aim in mind, we found it necessary to begin with a review of all available material on the subject for a more well-founded and encompassing view of the problem.

## Review

Methods

The PRISMA (Preferred Reporting Items for Systematic Reviews and Meta-Analysis) checklist [11] was used as a guideline to compile this systematic review.

An in-depth search of electronic databases such as PubMed and Google Scholar was conducted. Several keywords were used to locate relevant articles.

These were:

· "Impact of PPE and dermatological outcome”

· "Wearing PPE for a long time and dermatological manifestations"

· "Face mask-wearing and dermatological complications"

· "Wearing PPE and dermatological manifestations."

Inclusion criteria were as follows:

· Article must be dated between 2019 and 2021.

· Only complete articles were considered.

· Articles should be centered around using PPE for long periods, preferably with HCWs as the study sample (not mandatory).

**Figure 1:**
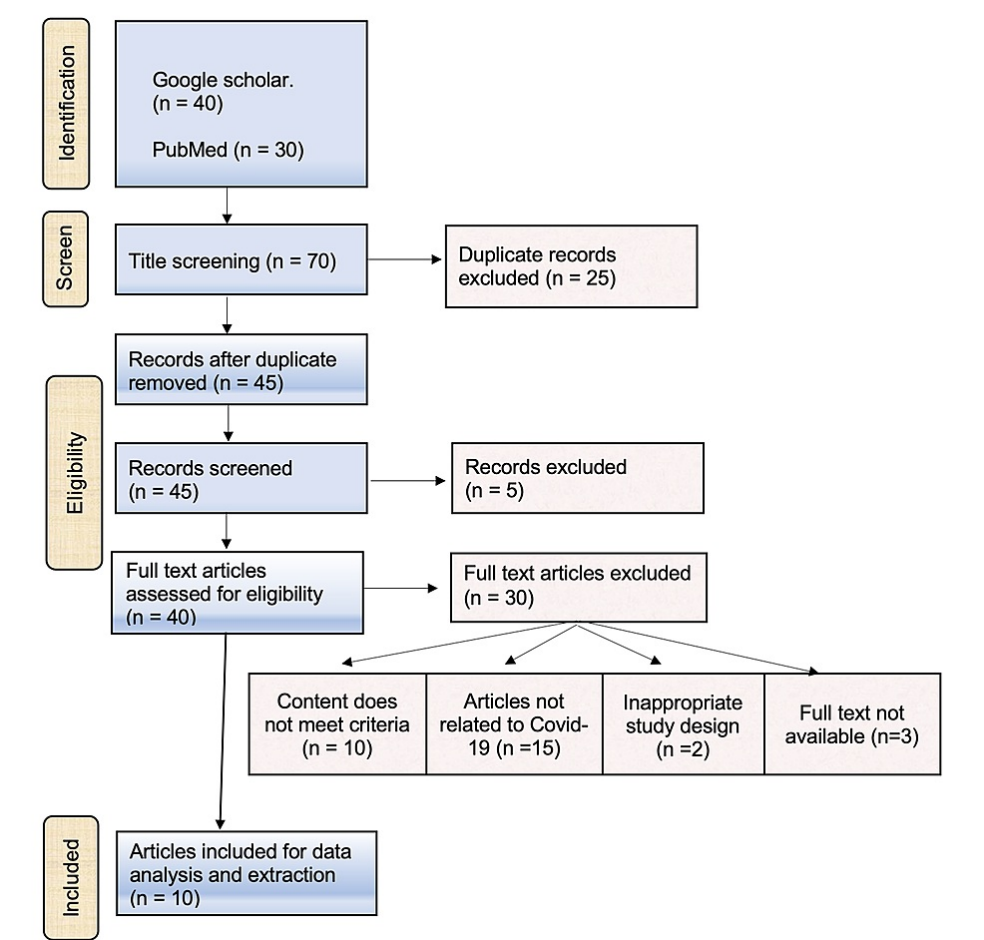
Planning of eligible criteria

A final review of the eligible documents was then carried out, with incomplete or overlapping studies being excluded further (Figure 1).

Data review and analysis

Stage one in the data review included a preliminary extraction of data via excel. This was proceeded by a more in-depth review in which research articles published by one research group examining similar variables were summarized in an attempt to prevent errors of duplication.

Results

Of the initial 70 articles, only 10 met the full inclusion criteria [12-21] (Table 1). The ten included studies varied slightly in their study designs; for example, seven of them were cross-sectional studies [12,13,15,18-21], one was a prospective study [14], and one was quantitative descriptive [17]. Only one study did not specify the used study design [16].

**Table 1:** Literature review of the 10 articles that met the full inclusion criteria PPE, Personal protective equipment; HCWs, healthcare workers; ASR, adverse skin reaction; HCP, healthcare professional; ADR, adverse drug reaction.

Author and Publication Year	Study Design	Population and Sample Size	Settings	Protective Tools Investigated and Duration	Results and Main Findings
Uthayakumar et al. 2021 [12]	Rapid report	- 67 staff members (nurses, doctors, pharmacists, radiographers, administrative, physiotherapy, laboratory technicians, domestic) - With age range of 23-60 years	London, UK	-	*Moderate eczema 91%, mild eczema 9%, moderate acne 33%, and mild acne 67%. *The most prevalent dermatoses were irritant contact hand dermatitis (45%), acne/folliculitis (24%), and eczema flare-up (28%). *One patient was diagnosed with Darier’s disease, exacerbated by PPE. *Hand eczema is one of the most common occupational diseases, and given the ongoing use of PPE and increased hand hygiene in both hospitals and public settings, we are likely to see an increase in these conditions, in both primary and secondary care. *With the use of PPE, there was a marked increase in the impact of skin conditions; 70% reported a significant adverse effect on their work or study, and 86% on well-being. *Dryness and associated irritation, in addition to pain and pruritus, were the commonly described symptoms and contributed to feelings of anxiety. *During COVID-19, the high prevalence of skin damage is in healthcare workers, which is corroborated in the findings from this study.
Purushothaman et al. 2021 [13]	Cross-sectional	- 250 healthcare workers - With an age of 20-48 years	SRM Medical College Hospital, Kattankulathur, India	- The use of N95 respirator and surgical face masks - Wearing duration of at least 4 hours/day	*Excessive sweating around the mouth 67.6%, difficulty in breathing on exertion 58.2%, acne 56%, itchy nose 52%, skin rashes/redness 39%. *The prolonged use of face masks induces difficulty in breathing on exertion and excessive sweating around the mouth to the HCWs, which results in poorer adherence and increased risk of susceptibility to infection.
Abiakam et al. 2021 [14]	*Prospective using a survey divided into distinct phases	- 108 staff of intensive care from a single center for the prevalence of adverse skin reactions during 24 of using PPE at different duration periods - 307 HCWs from different professional backgrounds	UK NHS centers	- Full protection PPE, eye protection, gloves, gown - The mean time of using PPE was 9.2 hours	*Adverse skin reactions with the bridge of the nose 96% and ears 30%. *The most common skin reactions included redness blanching 33%, itchiness 22%, and pressure damage 12%. *The use of PPE leads to an array of skin reactions at various facial locations of HCWs. *Modifications to PPE designs are required to accommodate a range of face shapes and appropriate materials to improve device safety. *Improvements in guidelines are required for PPE usage to protect skin health.
Techasatian et al. 2020 [15]	Prospective cross-sectional	- 833 participants of the general population (there were HCWs among participants) - Age of 18 years and older	Khon Kaen University, Faculty of Medicine, Thailand	- Four types of face masks: surgical (63.15%), cloth masks (30.05%), surgical masks covered by a piece of cloth (1%), N95 masks (0.72%)	*The prevalence of adverse skin reactions on the skin underneath the face masks was 54.5%. *The most frequent ASRs were acne (39.9%), rashes on face (18.4%), and itchy symptoms (15.6%). *HCW had adverse skin reactions related to face masks higher than non-HCW participants. *Wearing a surgical mask showed a higher risk of adverse skin reactions compared to wearing a cloth mask. *Wearing a face mask 4 to 8 hours/day and more than 8 hours/day increased the risk of adverse skin reactions on the face compared to wearing a face mask fewer or longer than 4 hours/day. *Not changing the mask after use every day was a risk factor showing 1.5 times the risk of having an adverse skin reaction.
Yuan et al. 2020 [16]	-	- 129 HCPs	Hospital of Zhejiang University School of Medicine, China	- L3PPE	*94.57% healthcare professionals experienced discomfort while wearing L3PPE to treat patients with COVID-19. *Adverse skin reactions including facial skin indentation, rash, and dermatitis. *Facial skin indentation was more prevalent among seniors (70.9%), rash was more prevalent among nurses (25.9%), and dermatitis was more prevalent among juniors (46.7%). *More attention should be offered to healthcare personnel wearing L3PPE to treat patients with COVID-19 because they are susceptible to developing adverse reactions.
Hu et al. 2020 [17]	Quantitative descriptive	- 61 HCWs	Hubei, China	- N95 masks, latex gloves, protective clothing	*The incidence of ADRs to the N95 mask was 95.1%, that to latex gloves was 88.5%, and that to protective clothing was 60.7%. *The most common ASRs among HCWs wearing N95 masks were nasal bridge scarring (68.9%) and facial itching (27.9%) after using N95 for 12 hours/day for 3.5 months. *The most common ADRs among HCWs wearing latex gloves were dry skin (55.7%), itching (31.2%), and rash (23.0%) with average usage of 10 hours for 3.5 months. *The most common ADRs among HCWs wearing protective clothing were dry skin (36.1%) and itching (34.4%). *The long-term use of PPE in Hubei Province found that most HCWs have adverse skin reactions when wearing masks, gloves, and protective clothing.
Jian et al. 2020 [18]	Multicenter, cross-sectional	- 4306 medical staff	161 hospitals in China	PPE levels 2 &3	*The overall prevalence of skin injuries was 42.8%; pressure injuries, moist-associated skin damage, and skin tear. *Sweating, daily wearing time more than 4 hours, male, and grade 3 PPE were associated with skin injuries. *The skin injuries among medical staff are serious, with insufficient prevention and treatment.
Coelho et al. 2020 [19]	Cross-sectional	- 1106 health professionals	- Ceara in Brazil	-	*The prevalence of pressure injuries related to the use of PPE was 69.4%, with an average of 2.4 injuries/professional. *The significant factors were age under 35 years, working and wearing PPE for more than 6 hours a day, in hospital units, and without the use of inputs for protection.
Shanshal et al. 2020 [20]	Cross-sectional observational	- 276 healthcare providers	- The major hospitals in Iraq	-	*The prevalence of hygiene-related hand dermatitis was 83%. *The prevalence of PPE-related dermatoses was 73.5%. *The types of PPE-related dermatoses included pressure injuries 51.9%, acne 33.1%, non-gloves CD 29.9%, non-specific rash 17.5%, urticaria 9.1%, and skin infections 3.2%.
Christopher et al. 2020 [21]	Cross-sectional	- 200 HCWs	- Siloam Hospitals, Indonesia	- PPE levels 2 & 3	*The prevalence of skin reactions was 66.5%; 73.7% females, 82.7% doctors, wearing levels 2 and 3 PPE 43.6% and 37.6%, respectively. *Most affected regions were cheeks and chin (69.9%). *The most common symptoms were dryness/tightness (63.9%) and acne (77.4%). *Sex, age group classification, level of PPE worn, hand hygiene frequency, and duration of PPE worn daily (≥7 hours) were factors considerably associated with adverse skin reactions to PPE. *Adverse skin reactions to PPE are common among HCWs in Indonesia.

The total number of participants in all 10 studies amounted to 7,643 participants; of these 833 (10.89%) were of the general population and the remaining 6,810 (89.1%%) were HCWs, both in private and public care sectors [15].

Concerning study locations, two were performed in the United Kingdom [12,14]; three studies in China [16-18]; and single studies from India [13], Thailand [15], Brazil [19], Iraq [20], and Indonesia [21]. There were three studies that did not specify the PPE investigated and used by the participants [12,19,20].

The included studies varied in the results and findings, so the finding of each study is explored individually. The rapid report, which included only 67 staff members, reported that eczema was the most common skin condition (91%), followed by irritant hand dermatitis (45%) and acne (33%). Hand eczema was the most common occupational disease due to the prolonged usage of PPE and increased hand hygiene. These skin reactions were found to affect the work of 70% of participants and affect the well-being of 86% [12].

A study on 250 HCWs investigated the wearing of N95 respirator and surgical masks for a period exceeding four hours daily and reported that acne was prevalent among 56% of participants, followed by nasal itching (52%), and skin rashes (39%). The prolonged usage of face masks affected the HCWs negatively, leading to poor adherence to legal obligations of constant usage of PPE and thus an increased risk of susceptibility to infection [13].

One study reported in the first phase that the prevalence of adverse skin reactions occurred around the nose (96%) and ears (30%); these were most commonly restricted to redness blanching as the main complication (33%), followed by itchiness (22%), and lastly pressure damage (12%). Moreover, the study reported that an average of 9.2 hours of constant exposure to PPE was necessary to exhibit these dermatological symptoms [14].

The study that was conducted on the general population reported a prevalence of 54.5% of adverse skin reactions on the skin underneath the face masks. The most common adverse reaction was acne (39.9%), followed by rashes (18.4%), and itchiness (15.6%). The study reported several risk factors for adverse skin reactions, including wearing a face mask for four to eight hours daily and not changing the mask after daily usage [15].

One study investigated the dermal impact of wearing level-3 PPE (L3PPE). It reported that 94.57% of healthcare professionals experienced discomfort related to wearing L3PPE. The most common adverse skin reactions were facial skin indentations, rashes, and dermatitis. In addition, skin indentation was more prevalent among seniors, whereas rashes and dermatitis were more prevalent among nurses and junior medical staff [16].

Another study that investigated the effect of N95 masks, latex gloves, and protective clothing by HCWs reported the incidence of adverse skin reactions based on the type of PPE in question. An increased incidence of adverse skin reactions was provoked by N95 masks (95.1%), followed by latex gloves (88.5%), and then protective clothing (60.7%). Nasal bridge scarring was the most prevalent skin manifestation due to wearing N95 (68.9%), whereas latex gloves and protective clothing resulted in dry skin (55.7% and 36.1%, respectively). These adverse skin reactions were found to be related to an exposure of at least 10 hours daily for 3.5 months [17].

The study that was conducted on the highest number of medical staff (4,306) and investigated the effects of level-2 and level-3 PPE reported a prevalence of skin injuries of 42.8%, with the main injuries being pressure injuries and skin damage associated with moist skin. Prolonged usage of PPE for more than four hours daily, being male, using level-3 PPE, and sweating were associated with skin injuries [18].

Another study that included 1,106 health professionals did not report specific investigated PPE; however, it showed that the prevalence of pressure injuries due to the use of PPE occurred among 69.4%, with an average of 2.4 injuries per practitioner tested. The occurrence of such injuries was associated with age (<35 years old), wearing PPE for more than six hours daily, no usage for inputs for protection, and hospital unit [19].

One study reported that the prevalence of dermatoses due to PPE was 73.5% and hand dermatitis was 83% [20]. These values are far higher when compared to previous studies, which reported that hand dermatitis was prevalent among only 45% of participants [12]. Pressure injuries and acne were the most prevalent dermatoses associated with PPE [20].

Another study investigating the impact of wearing level-2 and level-3 PPE reported a prevalence of skin reactions of 66.5%, of which 73.7% were females, 82.7% were doctors, and 43.6% of them had only worn level-2 PPE. The prolonged wearing of PPE for >seven hours, gender, age, the frequency of hand hygiene, and the level of PPE worn were associated with the adverse skin reactions among HCWs [21].

Discussion

The epidemiology of skin diseases related to masks has been rarely reported before, and most reports were case reports [10]. Nowadays, with the emergence of COVID-19 infection and the requirements for wearing PPE for a long time for both the general population and healthcare providers, there are several studies investigating the adverse skin reactions due to PPE. We included 10 studies to assess such dermatological manifestations, their prevalence, and risk factors increasing their incidence combined with the PPE.

The current lack of treatment for COVID-19 made the preventive measures such as wearing masks to be a crucial step to slow and prevent the transmission between the infected and healthy individuals [22]. However, the prolonged duration of mask contact could increase the burden of allergic contact dermatitis [23].

In this systematic review, we could identify several dermal conditions associated with wearing PPE as reported in different studies from different regions in the world. The prevalence of the dermatological adverse reaction varied from 42.8% to 95.1% [17,18]. The prevalence of such conditions and their types vary between the different studies included, and this returns to the variation in the study populations and their skin susceptibility, the time investigated for the usage of PPE, and the risk factors. However, we could identify that eczema, especially hand eczema, acne, skin rashes, itchy symptoms, and pressure injuries, were the most common dermatological complications of wearing PPE.

A case of a 32-year-old nursing male suffering occupational contact dermatitis on his face on a background of mild acne, which was found to be related to dibromo-dicyanide-butane released from the recently introduced surgical mask was published in 2017 before the outbreak of COVID-19. Additional proof of the correlation between the use of mask and his condition was the significant improvement of the clinical presentation of the patient after avoiding the use of the mask [24].

The included studies investigated the impact of wearing PPE for different durations including, ≥ four hours, ≥ seven hours, or a mean of 9.2 hours daily. The adverse skin reactions were significantly more apparent among individuals wearing PPE for ≥ four hours daily. Also, being a healthcare provider made the individual more prone to suffer adverse skin reactions compared to the general population; this can be related to the long duration the healthcare providers spent in the healthcare setting. Moreover, the position of the healthcare provider was also a determinant factor for the incidence of adverse skin reactions, where seniors were more prone to suffer dermatological complications compared to juniors, and both were more prone to suffer dermatological complications compared to nurses [16].

The prevalence and the type of adverse skin reactions were affected by the PPE type and level; it was found that wearing N95 was associated with a higher incidence of adverse skin reactions [17]. Also, wearing the surgical mask was a risk factor for developing adverse skin reactions compared to wearing a cloth mask [15]. Though level III PPE was associated with discomfort [16] and skin injuries [18], a study reported that level two PPE resulted in a higher frequency of skin reactions (43.6%) compared to level III PPE (37.6%) [21]. The type of skin reaction and complication was affected by the type of protective measures used; wearing N95 masks was associated with nasal bridge scarring and facial itching, whereas latex gloves resulted in dry skin, itching, and rashes, and wearing protective clothing resulted in dry skin and itching [17].

The hygiene and its frequency were associated with the incidence of dermal complications. Hand eczema was one of the most common occupational dermatological diseases due to the usage of PPE and increased hand hygiene in the hospital and public settings [12]. Moreover, hygiene-related hand dermatitis was found to occur among 83% of healthcare providers [20].

All of these adverse skin reactions and complications that occurred due to the use of PPE made authors suggest that they can result in poor adherence and hence increase the risk for infection [13]. Therefore, it was suggested that the design of PPE should be modified to accommodate a range of face shapes and materials to improve the safety of the protective tool and to be gentle on the skin. Also, the guidelines can be improved for the usage of PPE to protect skin health [14].

Another study suggested using virtual occupational health checks to prevent serious skin damage among healthcare providers [25]. Hydrocolloid dressings have been suggested to be used in order to improve comfort and reduce skin damage among those using facial masks [26]. The use of hydrogel patches was found to reduce the skin reaction by almost 10 times compared to the non-users [27]. Regarding pressure injuries, the use of a correctly fitted mask can reduce friction.

## Conclusions

Dermatologic complications are more common due to the usage of PPE or face masks during the COVID-19 pandemic. The most commonly reported skin conditions were eczematous eruptions, acne, erythematous rashes, dry hand skin, and pressure injuries such as nasal bridge scaring. The length of exposure time and the magnitude of protective measures used have been linked to the onset and severity of these cutaneous complications; healthcare providers who wore full PPE for longer durations reported a higher incidence of dermatological complications compared to the general population. Moreover, a higher professional ranking, like seniors, and an older age have been associated with increased risk of side effects from PPE among HCWs, which could be explained by their increased responsibilities and longer exposure time. On the other hand, subjects of the general population suffered mostly from dry hands, rashes around the ears, and acne along mask borders. Increased contact time and infrequent change of face masks were significant risk factors for the development of these skin complications in the general population. These adverse skin reactions to PPE and face masks may lead to poor adherence to their use, thus increases the possibility of unprotected exposure to the virus. Therefore, using the proper type of protective measures, minimizing the duration of exposure, and replacing the used masks frequently are all recommended to avoid adverse skin reactions and to optimize adherence.
